# A conversation with Yuk Ming Dennis Lo

**DOI:** 10.1172/JCI166777

**Published:** 2022-12-15

**Authors:** Ushma S. Neill

The winner of the 2022 Lasker~DeBakey Clinical Medical Research Award, Yuk Ming Dennis Lo is a physician-scientist at the Li Ka Shing Institute of Health Sciences of the Chinese University of Hong Kong. Lo (Figure 1) is often called the father of noninvasive prenatal testing. After discovering fetal DNA in maternal blood, Lo catalyzed a medical revolution that has saved millions of pregnant people from having to undergo invasive tests like amniocentesis. With Lo’s pioneering technical advances, those who are pregnant can easily and reliably be screened for Rh factor mismatch, trisomies, and genetic disorders, and the implications of his work have and may go further in cancer testing, transplantation, and perhaps beyond. For the full interview, see https://www.jci.org/videos/cgms.

*JCI*: What were you like as a child?

Lo: I was born in Hong Kong; my father was a psychiatrist, and my mother was a music teacher. My mom used to tell me that I was very naughty as a child, keeping the whole family up all night. When I went to kindergarten, somehow, I got into a girls’ school; the whole class was forty girls and three of us boys. I was perhaps a bit more active than the others, and every time a report card came home, I would get a C– in conduct. Academically, during my initial years at primary school, I was only OK, but towards the end of primary school, my grades got better.

At the time, the number of university places in Hong Kong was very low — I think about 10% of us could manage to get into university. I needed to apply internationally as well, but I had never been to Europe or the US, so it was a bit frightening. In my secondary school, we had a biology textbook that had pictures of famous scientists, and one of the pictures was of Watson and Crick standing in front of King’s College Chapel at Cambridge, and I thought that looked like a very nice place, so I applied to Emmanuel College, Cambridge, and was accepted. Cambridge has a tutorial system in which there’s one supervisor looking after two to three students. It was quite daunting, writing essays in my nonnative language and then reading them sentence by sentence to the supervisor. Recall I’d never been to the UK before, so I had to get used to all the different accents — maybe understanding only 60% of what anyone said.

*JCI*: You moved to Oxford for your medical degree.

Lo: During one of my trips to Oxford, I visited a college called Christ Church. I was very impressed. Christ Church later became the model for Hogwarts from the Harry Potter movies. When I went to Oxford, I did get into Christ Church; that college had a long medical tradition. At that time, I thought I might pursue medical practice, but on the other hand, my education at Cambridge was very scientific. I didn’t have a fixed idea what I wanted to do, so I engaged in some research on the side that eventually changed my career. There was a senior research fellow by the name of John Bell who had just come back from Stanford to Oxford. Now known as Sir John Bell, he was one of the first to publish on PCR and he told us in a lecture that PCR was going to change the world. As a young student, I was fascinated, so after the lecture, I asked him to teach me how to do PCR, which he did. That was around 1987, and doing PCR was much more complicated than it is now — there was no Taq polymerase, so every cycle, I had to add enzyme and it was very nonspecific. Also, there were no thermocyclers, so everything was manual — PCR demanded three to four hours sitting in front of a bench moving test tubes between water baths. There were also a lot of false-positive results due to contamination.

During clinical training in obstetrics, I learned about the Kleihauer-Betke test that determines if there is fetal blood in the maternal circulation to assess for fetal hemorrhage. I thought PCR could work better, since most adult red cells don’t have a nucleus, but fetal cells do. As a first try, we did a PCR experiment to find Y chromosomes in the maternal blood cells and we were able to find a signal. It showed that, in theory, one could detect fetal cells. One of the challenges is that the number of fetal cells is extremely low. I spent years chasing these rare cells, and by the time I graduated with a DPhil in ‘94, I didn’t have a method that was even remotely clinically applicable. My scholarship was finishing; I had to face reality and went back into clinical practice. Gratifyingly, the Wellcome Trust had just started a career development fellowship that armed me with a lab technician. Before I went on rounds, I’d talk with the technician, and then she’d do the research work, and after my clinical duty, I’d come back to discuss. At that juncture, the scientific infrastructure just was not ready for the kind of work I would eventually be doing; there was no reference human genome, no massively parallel sequencing. That period, something like eight years, although slow, nonetheless paved the way for my subsequent work.

*JCI*: Near the end of this time, you and your wife decided to move back to Hong Kong to take up your next academic posts.

Lo: My wife and I were both born in Hong Kong, and our parents were there; 1997 was the year that Hong Kong went back to the sovereignty of China, and some faculty members chose to leave. Both of the medical schools in Hong Kong were recruiting in my area, and happily, I got a post. However, I only got about six feet of a bench and one assistant and also had significant clinical and teaching duties; that was a challenging time. I knew that I had to start from scratch and wanted to try something more innovative and unusual. For the previous eight years, I worked on circulating cells, but now turned to blood and plasma. Scientifically, it was a very exciting time. I remember when we first looked at maternal plasma and saw that we could detect cell-free DNA, I could not believe my eyes. All these years, we were looking in the wrong place. It was just amazing that we could take a few drops of plasma and boil it for five minutes and get a signal that was so robust.

*JCI*: Did you quickly recognize what the implications would be for prenatal testing?

Lo: Initially we used the Y chromosome as a marker; if a pregnant mother was carrying a boy, we could see the Y chromosome. Later, we did blood groups and specifically rhesus positive and negative. Imagine the scenario of a mother who is rhesus negative and a baby is positive: we could detect that and trigger early treatment. We moved on to detecting some genetic diseases that have multiple mutations and especially those where the mutations come from the father. Those were low-hanging fruit. But then for Down syndrome, we knew it would be challenging, as the frequency of Down syndrome increases with the mother’s age, meaning most of the time, the extra chromosome 21 is from the mother. Differentiating the extra fetal chromosome 21 from the mother’s own was a challenge. What we didn’t anticipate is that it would take ten years to crack that nut.

*JCI*: How involved were you in the discussions centered on the ethical implications of your work?

Lo: We’ve always been very conscious about the ethical implications of our research. In the licensing clause for using our patents, we stipulate the technology cannot be used for selective abortion because of sex. With regard to genetic maladies or chromosomal trisomies, prenatal testing should only be done together with counseling. Without our technology, pregnant women who are already doing prenatal testing are engaging in testing that is not very accurate. Nuchal translucency and maternal serum screening yield that 5% of the women tested will actually be classified as being at high risk. On the other hand, we know Down syndrome incidence is 1 in 800. So in most cases, the baby is normal, and the mother and baby are often unnecessarily subjected to an invasive procedure, which carries risk. Our technology is meant to save the baby and mother from that process.

*JCI*: You also took on trying to elaborate the entirety of the fetal genome.

Lo: As we were contemplating what we were going to do next, we knew the goal was deciphering the entire fetal genome. Eventually, we deduced that we needed two formulas: one for the father’s side and one for the mother’s side. The idea came to me as I was watching a 3D (the first 10 minutes or so) showing of *Harry Potter and the Half-Blood Prince*. The H of the Harry Potter logo — the two strokes looked to me like a pair of chromosomes. One copy is from the father, the other one is from the mother, so maybe we need two algorithms. And that was how we cracked it.

*JCI*: Do you think that the ethics around knowing the whole fetal genome are somewhat different?

Lo: We published the technique in 2010 and made it onto the cover of *Science Translational Medicine*. On that day, we had a lot of media coverage, much of which discussed the ethical implications. With whole-genome sequences, you can detect any genetic disorder. But you can also potentially detect a variety of other genetic variations in which you are not so sure of the implications, or you might even detect diseases that are late onset. Let’s say that if you see something which tells you a baby might have diabetes or something that might develop when they’re over 40, what would you do?

The consensus is that if you use this technology, it should only be for genetic disorders that are very serious or even lethal rather than for detection of diseases that may occur in the future. In the future, if we develop the means to treat some of those maladies, then maybe that will change.

Let me give you one example, a condition called congenital adrenal hypoplasia (CAH), in which a baby has a genetically driven endocrine disorder that causes them to produce excess androgens — more male sex hormones than usual. If this happens in a baby boy, the outcome may be less severe. But if it happens in a baby girl, she’ll be born with virilized external genitalia. Subsequently, that baby might be subjected to numerous surgeries. With prenatal fetal-genome sequencing, if we knew that a baby had CAH even in utero at eight weeks of development, we could give the mother dexamethasone and the baby could develop normally.

*JCI*: The next frontier that you have taken on with cell-free DNA is cancer detection.

Lo: A baby with a placenta growing into the uterus is a little bit similar to a tumor that invades into tissue. So it opens the possibility that whatever we found on the fetal side could also be applied in cancer. In the fetus, the low-hanging fruit was detection of the Y chromosome, and so we looked for the low-hanging fruit in cancer. Some cancers are associated with viral infection; nasopharyngeal cancer (NPC) is one such example. This is a cancer at the back of a throat and is particularly common in China, and a man like me in that area has a lifetime likelihood of developing NPC of 1 in 39, usually occurring between 40 and 60 years old.

I hypothesized we could use the causative virus, Epstein-Barr virus (EBV), as a similar marker to the Y chromosome. EBV is very common in the adult population — maybe 99% of us are chronic carriers of EBV. When I first proposed this idea, many clinicians laughed at me, predicting we’d have plentiful false-positive results. Interestingly, in a normal, healthy individual, EBV DNA is *inside* cells, but in cancer, the DNA is *outside* cells. We showed that detecting cell-free EBV DNA was a pretty good test to detect whether someone had NPC. Levels increased at later stages, and upon successful treatment, levels dropped; if the cancer relapsed, levels came back up.

We were able to study 20,000 subjects, screened those individuals, and discovered 34 cases of NPC. Normally, by the time you detect NPC, 75% to 80% are late stage, but now with screening, we were able to shift to finding 70% at the early stages, wherein the chance of survival is 10 times higher.

*JCI*: If you could not have been a physician or a scientist, what other career do you think could have captivated you?

Lo: I have always tried to be precise with the way I write and the way I present arguments. Given my experience with patenting our work, I think an alternative to being a scientist would be to become a lawyer and likely a patent lawyer.

## Figures and Tables

**Figure 1:**
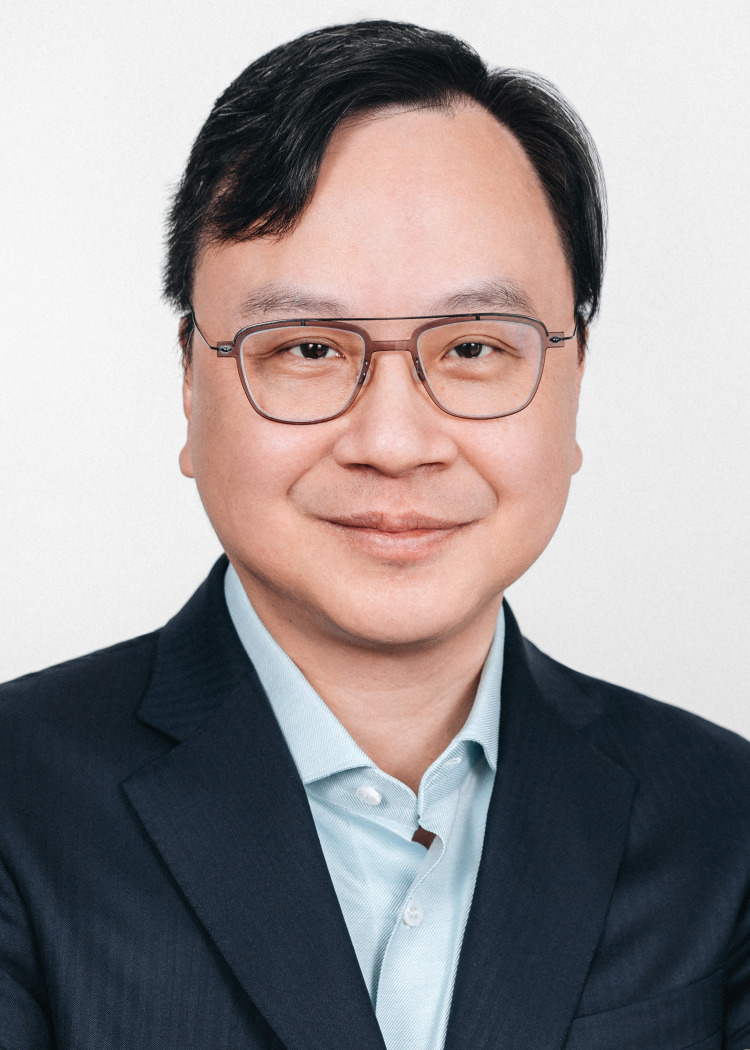
Dennis Lo in New York City on September 23, 2022. Image credit: CEOportrait.com.

## Version 1 12/15/2022

Electronic publication

